# Tau passive immunization inhibits not only tau but also Aβ pathology

**DOI:** 10.1186/s13195-016-0227-5

**Published:** 2017-01-10

**Authors:** Chun-ling Dai, Yunn Chyn Tung, Fei Liu, Cheng-Xin Gong, Khalid Iqbal

**Affiliations:** Department of Neurochemistry, New York State Institute for Basic Research in Developmental Disabilities, Inge Grundke-Iqbal Research Floor, 1050 Forest Hill Road, Staten Island, NY 10314 USA

**Keywords:** Alzheimer’s disease, Amyloid-β, Immunotherapy, Tau, Tauopathy

## Abstract

**Background:**

Accumulation of hyperphosphorylated tau protein is a histopathological hallmark of Alzheimer’s disease (AD) and related tauopathies. Currently, there is no effective treatment available for these progressive neurodegenerative diseases. In recent years, tau immunotherapy has shown great potential in animal models. We report the effect of immunization with tau antibodies 43D against tau 6–18 and 77E9 against tau 184–195 on tau and amyloid-β (Aβ) pathologies and cognition in triple-transgenic (3×Tg)-AD mice at mild to moderate stages of the disease.

**Methods:**

We immunized 12-month-old female 3×Tg-AD mice with two to six or seven intravenous weekly doses of 15 μg of mouse monoclonal antibody 43D, 77E9, a combination of one-half dose each of 43D and 77E9, or as control of mouse immunoglobulin G (IgG). Age-matched wild-type mice treated with mouse IgG or a mixture of 43D and 77E9 were also used as controls. The effect of immunization with tau antibodies on tau and Aβ pathologies was assessed by Western blot and immunofluorescence analysis, and the effect on cognition was analyzed by using Morris water maze, one-trial novel object recognition, and novel object location tasks.

**Results:**

We found that two doses of 43D and 77E9 reduced total tau but had no significant impact on hyperphosphorylation of tau. However, six doses of 43D reduced levels of both total tau and tau hyperphosphorylated at Ser262/356 and Ser396/404 sites in the hippocampus. Importantly, both 43D and 77E9 antibodies rescued spatial memory and short-term memory impairments in 3×Tg-AD mice. The beneficial effect of 43D and 77E9 antibodies on cognitive performance was sustained up to 3 months after the last dose. Six doses of immunization with 43D also decreased amyloid precursor protein (APP) level in CA1 and amyloid plaques in subiculum, and showed a trend toward reducing Aβ40 and Aβ42 in the forebrain. Immunization with 43D increased levels of complement components C1 and C9 and resulted in activation of microglia, especially surrounding Aβ plaques.

**Conclusions:**

These findings suggest the potential of passive immunization targeting proximal N-terminal domain tau 6–18 as a disease-modifying approach to AD and related tauopathies.

## Background

Alzheimer’s disease (AD), which is characterized by progressive loss of memory and other cognitive functions, is the most common cause of dementia. AD is the sixth leading cause of death in the United States [1]. The two major histopathological hallmarks in brains of patients with AD are extracellular senile plaques consisting of amyloid-β (Aβ) peptides [2] and intracellular neurofibrillary tangles (NFTs) composed of abnormally hyperphosphorylated tau protein [3]. The tau pathology made up of the hyperphosphorylated tau is also a hallmark of several neurodegenerative disorders known as *tauopathies*, including frontotemporal dementia, corticobasal degeneration, progressive supranuclear palsy, Pick disease, parkinsonism-dementia complex of Guam, and chronic traumatic encephalopathy. Currently, there are no effective treatments available for AD and related tauopathies. Based on an Aβ cascade hypothesis [4], most therapeutic approaches including Aβ immunotherapy for AD are focused mainly on reducing Aβ plaques in the brain [5, 6]. To date, none of the treatments available for AD slow or stop neurodegeneration. Clinical trials with Aβ therapies, including immunotherapy, have thus far failed to show any reduction in neurofibrillary pathology or improvement in cognitive performance of patients with AD [5–10].

Multivariate analyses indicate that the density of NFTs, neuron number loss, and synapse loss, but not amyloid load, strongly correlates with cognitive impairment in patients with AD [11–14]. Thus, targeting tau pathology could be clinically more efficacious than Aβ-directed clearance therapy for patients with AD. Indeed, active and passive immunotherapies targeting pathological tau have been tested in several transgenic mouse models of AD using different phospho-tau peptides and antibodies, and they have shown great potential [15]. Active immunization with tau phosphopeptides reduces tau pathology [16–22] and rescues or slows cognitive decline in rodents [16, 19, 21, 22]. Passive immunotherapy using antibodies against tau has also been shown to slow disease progression [19, 23–28].

Based on the success of tau immunization in transgenic mouse models of overexpression of disease-causing mutated taus, tau immunotherapy is becoming a rapidly growing area of drug development in clinical trials. A phase I study of the first tau immunotherapy targeting active immunization against tau 294–305 in patients with AD has been completed, and recruitment of patients for a phase II human clinical trial is ongoing (ClinicalTrials.gov identifier: NCT02579252; AXON Neuroscience SE, Bratislava, Slovak Republic). ACI-35 is a liposome-based vaccine targeting tau 393–408 that has been tested in a completed phase I study and is now being evaluated in a phase Ib trial of patients with mild to moderate cases of AD [29]. More recently, additional clinical trials targeting passive immunization were initiated, which include one against N-terminal fragment of tau (tau 17–28; BMS-986168/IPN007) by Bristol-Meyers Squibb (ClinicalTrials.gov identifier: NCT02294851; BMS-986168) and another against tau pSer422 (tau 416–430; RO6926496) by Hoffman-La Roche (ClinicalTrials.gov identifier: NCT02281786). In addition, a phase I study with C2N-8E12 antibody in patients with progressive supranuclear palsy (ClinicalTrials.gov identifier: NCT02494024; C2N Diagnostics) is ongoing.

Previously, we discovered that abnormally hyperphosphorylated tau from patients with AD, instead of interacting with tubulin and promoting its assembly into microtubules, sequesters normal tau, forming oligomers and consequently filaments that can be sedimented at 100,000–200,000 *g* [30–32]. This finding, which formed the basis of tau spread studies [33–36], led us to test the removal of pathological tau by passive immunization using antibodies to N-terminal domains of tau.

Tau pathology is well documented to propagate in a predictable pattern [37, 38]. The abnormally hyperphosphorylated/oligomeric tau released in the extracellular space from the affected neurons is suspected to serve as the seed for the spread of tau pathology by the ingesting cells [33, 36, 39, 40]. Tau immunotherapy may clear extracellular tau that is involved in the spreading of tau pathology [23, 41].

In a previous study, we discovered that passive immunization with tau antibody to the N-terminal domain of tau not only reduced tau pathology but also showed a trend toward ameliorating Aβ pathology in triple-transgenic (3×Tg)-AD mice at moderate to severe stages of the disease [28]. In the present study, we administrated tau antibodies by intravenous injection into 3×Tg-AD mice at a mild stage of the pathology (12 months old) once weekly for up to 6 weeks; 3×Tg-AD mice start developing Aβ plaques at approximately 9 months and tau pathology starts at approximately 12 months [42–44]. We found that passive immunization with monoclonal antibody 43D against tau 6–18 could reduce not only tau but also Aβ pathology in 3×Tg-AD mice and that the N-terminal proximal domain of tau was a more effective target for passive immunization than the N-terminal distal domains of tau.

## Methods

### Antibodies and reagents

Primary antibodies used in this study are listed in Table 1. Peroxidase-conjugated antimouse and antirabbit immunoglobulin G (IgG) were obtained from Jackson ImmunoResearch Laboratories (West Grove, PA, USA). We used an enhanced chemiluminescence kit from Pierce Biotechnology (Rockford, IL, USA). Human Aβ_1–40_ and human Aβ_1–42_ enzyme-linked immunosorbent assay (ELISA) kits were obtained from Invitrogen (Carlsbad, CA, USA). Modified Dulbecco’s PBS buffer was obtained from Thermo Fisher Scientific (Waltham, MA, USA). Other chemicals were purchased from Sigma-Aldrich (St. Louis, MO, USA).

**Table 1:** Primary antibodies used in this study

Antibody	Type	Specificity	Phosphorylation sites	Source/reference
43D^a^	Monoclonal	Tau		Covance (Princeton, NJ, USA)
77E9^a^	Monoclonal	Tau		Covance
R134d	Polyclonal	Tau		[77]
GAPDH	Polyclonal	GAPDH		Santa Cruz Biotechnology (Dallas, TX, USA)
pS199	Polyclonal	p-tau	Ser199	Invitrogen (Carlsbad, CA, USA)
AT8	Monoclonal	p-tau	Ser202/Thr205	Thermo Fisher Scientific (Waltham, MA, USA)
pT205	Polyclonal	p-tau	Thr205	Invitrogen
12E8	Monoclonal	p-tau	Ser262/356	Dr. D. Schenk, Elan Pharmaceuticals (South San Francisco, CA, USA)
PHF1	Monoclonal	p-tau	Ser396/404	Dr. P. Davies, Albert Einstein College of Medicine (Bronx, NY, USA)
APP	Polyclonal	APP		Generated in our institute
4G8	Monoclonal	APP/Aβ		BioLegend (San Diego, CA, USA)
Iba1	Polyclonal	Iba1		Abcam (Cambridge, MA, USA)
Iba1	Polyclonal	Iba1 for IHC		Wako Chemicals (Richmond, VA, USA)
C1q	Monoclonal	C1q		Thermo Fisher Scientific
C9	Polyclonal	C9		Thermo Fisher Scientific

*Abbreviations: APP* Amyloid precursor protein, *Aβ* Amyloid-β, *GAPDH* Glyceraldehyde 3-phosphate dehydrogenase, *IHC* Immunohistochemistry, *PHF1* Paired helical filament 1, *pS199* Phospho-tau (Ser199), *pT205* Phospho-tau (Thr205), *p-tau* Phosphorylated tau

^a^Immunoglobulin G1 kappa light chain

### Immunization of animals

Mouse monoclonal tau antibodies 43D against tau 6–18 and 77E9 against tau 184–195 were generated at our institute. Both 43D and 77E9 were IgG1 kappa light chain. Briefly, recombinant human tau 441 purified from *Escherichia coli* was used as the immunogen. Tau was emulsified in the presence of complete Freund’s adjuvant (1:1 vol/vol; Difco Laboratories, Detroit, MI, USA) with the minichanger of an Omni Mixer (Omni International, Kennesaw, GA, USA) at 4 °C. BALB/cJ mice (7 weeks old; The Jackson Laboratory, Bar Harbor, ME, USA) were inoculated intradermally and subcutaneously with tau as previously described [45]. The animals were bled retro-orbitally and monitored for antibody response by the ELISA procedure.

### Production of hybridomas

Spleen cells were fused to the NS0 myeloma cell line. Initial screening of hybridoma supernatants from 96-well plates was performed by ELISA. Each selected hybridoma was cloned three times by limiting dilution and used to produce ascites in 2,6,10,14-tetrametylpentadecane (Pristane; Sigma-Aldrich)-primed mice. An Ig subtype identification kit (Boehringer Mannheim Biochemical, Indianapolis, IN, USA) was used for isotyping the antibody.

### Epitope mapping

Epitope mapping of each tau antibody was carried out using 12-mer overlapping synthetic tau peptide manufactured by Pepscan (Lelystad, The Netherlands).

### Mice

The homozygous 3×Tg-AD mice harboring human APP_SWE_ and tau_P301L_ transgenes with knock-in PS1_M146V_ under the control of the mouse Thy1.2 promoter, created in the laboratory of Dr. Frank LaFerla [42], were obtained from The Jackson Laboratory (https://www.jax.org/strain/004807). Homozygous male and female 3×Tg-AD mice on the mixed C7BL/6;129X1/SvJ;129S1/Sv genetic background were bred in the animal colony of New York State Institute for Basic Research in Developmental Disabilities (Staten Island, NY, USA). Mice had access to food and water ad libitum and were housed (four or five animals per cage) in pathogen-free facilities with 12-h light/12-h dark cycles. The female 3×Tg-AD mice develop amyloid plaques starting at about 9 months of age and NFTs starting about 12 months of age, respectively, and the pathologies are predominantly restricted to the hippocampus, amygdala, and cerebral cortex [42–44].

B6129SF2/J strain mice, used as wild-type (WT) controls in the present study, were the offspring of a cross between C57BL/6J females (B6) and 129S1/SvImJ males (129S); they are commonly used as controls for genetically engineered strains generated with 129-derived embryonic stem cells and maintained on a mixed B6;129 background (https://www.jax.org/strain/101045).

To avoid the effect of the variations among the mice on the results, at the beginning of the present study, all mice used were first grouped according to their body weight and age, and the mice from the same litter were evenly assigned to different groups. Then the grouped mice were randomized into (1) WT mice treated with IgG, (2) WT mice treated with tau antibodies 43D + 77E9, (3) 3×Tg-AD mice treated with IgG, (4) 3×Tg-AD mice treated with 43D, (5) 3×Tg-AD mice treated with 77E9, and (6) 3×Tg-AD mice treated with 43D + 77E9.

### Immunization with tau antibodies

To investigate the dose-dependent effect of passive immunization with N-terminal tau antibodies on reduction of tau pathology, we immunized the 3×Tg-AD mice (four or five mice per group) intravenously with 15 μg of 43D, 77E9, and as controls with mouse IgG in 200 μl of saline once per week for 2 or 6 weeks. Animals were killed 1 day after their last immunization.

**Fig. 1:**
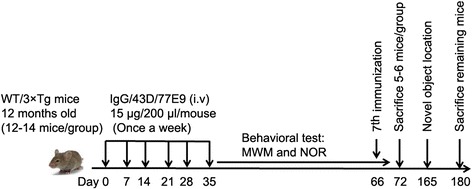
Study design. 3×Tg-AD mice were subjected to Morris water maze and one-trial novel object recognition tasks after immunization with six doses of mouse IgG (control), 43D, 77E9, and combination (50% each) of 43D and 77E9 antibodies. On day 66, the mice were administrated one more dose; five or six mice per group were killed on day 72; and the remaining animals were killed on day 180. The novel object location test was carried out from day 165 to day 167. WT mice immunized with mouse IgG or a combination of 43D and 77E9 were used as controls. *AD* Alzheimer’s disease, *IgG* Immunoglobulin G, *MWM* Morris water maze, *NOR* Novel object recognition, 3×*Tg* Triple-transgenic, *WT* Wild type

To investigate the effect of 43D and 77E9 on cognition, 12-month-old female mice were used, and behavioral tests were carried out 1 day after the sixth immunization. After behavioral tests, animals received one more dose on day 66, and five or six mice per group were killed on day 72. The remaining mice were housed for another 4 months. A one-trial novel object location test was carried out before these mice were killed (Fig. 1).

After removal of brain, one-half of the brain was fixed in 4% paraformaldehyde for histological and immunohistological studies, and the other half was dissected into forebrain including hippocampus and stored at −80 °C for biochemical analysis.

### General examination

A general examination of all the mice was conducted in the home cages throughout the whole study. Any gross abnormalities in overall health, home cage nesting, sleeping, feeding, grooming, and condition of the fur of animals were noted. Body weight was measured once per week during the study period.

### Morris water maze task

The Morris water maze task was used to evaluate spatial learning and memory of the mice [46, 47]. A total of 74 mice were subjected to the Morris water maze task from 1 day after the sixth immunization. The test was performed in a circular white pool (with a diameter of 180 cm and a height of 60 cm) filled with nontoxic white dye-tinted water and maintained at room temperature (20 ± 1 °C). The maze was designed with two virtual principal axes, with each line bisecting the maze perpendicular to the other one to divide the maze into four equal quadrants. The end of each line demarcates four cardinal points: north, south, east, and west. A platform was positioned in the middle of one of the quadrants submerged 1 cm below the water surface. Each mouse performed two trials on day 1 and 2, three trials on day 3, four trials on days 4 and 5 for 5 consecutive days from semirandom start positions [48] to find the hidden platform. Each trial was terminated as soon as the mouse climbed onto the hidden platform. If a mouse failed to find the platform within 90 seconds, it was gently guided to the platform. At the end of each trial, the mouse was left on the platform for 20 seconds, then removed, dried, and returned to its home cage. A 90-second probe test without platform was performed 24 h after the last trial. Escape latency (seconds) in initial training as well as latency to first entrance into target (platform location area), target crossings, swim speed (centimeters per second), and time spent in each quadrant (seconds) in the probe test were recorded through an automated tracking system (Panlab SMART video tracking system version 2.0.14; Harvard Apparatus, Holliston, MA, USA).

### One-trial novel object recognition task

The one-trial object recognition test is based on the innate tendency of rodents to explore novel objects over familiar ones and is believed to measure episodic memory. The experimental procedure was performed as described previously [49, 50]. Testing consisted of three different phases: a habituation phase, a sample phase, and a test phase. Following initial exposure, four additional 10-minute daily habituation sessions were performed for mice to become familiar with the apparatus (50 × 50 × 40 cm) and the surrounding environment. On the fifth day, every mouse was first subjected to the sample phase, in which two identical objects were placed in a symmetrical position from the center of the arena and the mouse was allowed to freely explore the objects for 8 minutes. After a 20-minute delay during which the mouse was returned to its home cage, the animal was reintroduced into the arena to perform the test phase. The mouse was then exposed to two objects for another 5 minutes: a familiar object (previously presented during the sample phase) and a novel object, placed at the same location as during the sample phase. Data collection was performed using a video tracking system (ANY-maze version 4.5 software; Stoelting Co., Wood Dale, IL, USA). The object discrimination index was evaluated by the index [(time spent exploring the new object)/(time spent exploring both old and new objects) × 100%] during the test phase.

### Novel object location task

The object location memory task has been used to evaluate hippocampus-dependent spatial memory in rodents through an evaluation of the ability of rodents to explore the new location of a familiar object with respect to spatial cues [51]. Testing was conducted in an open-field apparatus (50 × 50 × 40 cm). The mice were first acclimated to the chamber for 10 minutes per day for 2 days. On the third day during the acquisition phase, mice were allowed to explore two identical objects, which were placed in the far corners of the arena for 8 minutes. After a delay of 20 minutes, one object was placed in the diagonally opposite corner. Thus, both objects in the test phase were equally familiar, but one was in a new location. The location of one of the two objects was changed at random but counterbalanced for each mouse. The objects were cleaned with 70% ethanol after each trial. The time exploring each object was recorded. A discrimination index was calculated as [(new location object exploring time)/(time spent exploring both familiar and new location objects) × 100%] during the test phase [52, 53].

### Western blot analysis

Mouse brain tissue was homogenized in prechilled buffer containing 50 mM Tris-HCl, (pH 7.4), 100 mM sodium fluoride, 1 mM sodium orthovanadate, 1 mM ethylene glycol-bis(2-aminoethylether)-*N,N,N′,N′*-tetraacetic acid (EGTA), 0.5 mM 4-(2-aminoethyl)benzenesulfonyl fluoride hydrochloride (AEBSF), 10 μg/ml aprotinin, 10 μg/ml leupeptin, and 4 μg/ml pepstatin. Each homogenate was boiled in 2× Laemmli sample buffer for 5 minutes, and protein concentration was measured by Pierce^TM^ 660-nm protein assay (Thermo Scientific, Rockford, IL, USA). The samples were resolved by 10% SDS-PAGE and electrotransferred onto Immobilon-P membranes (EMD Millipore, Billerica, MA, USA). The blots were then probed with primary antibodies (Table 1) and developed with the corresponding HRP-conjugated secondary antibody and enhanced chemiluminescence kit (Pierce Biotechnology). Densitometric quantification of protein bands in Western blots was analyzed using Multi Gauge version 3.0 software (FUJIFILM North America, Valhalla, NY, USA).

### Immunohistochemical staining

Immunohistochemical quantification of abnormally hyperphosphorylated tau was performed on three or four sections from three to five mice per group. Free-floating sagittal sections were washed in 10 mM PBS (15 minutes, three times each) and then incubated in 0.3% Triton X-100 for 30 minutes. The sections were again washed in 10 mM PBS (15 minutes, three times each) and blocked in blocking solution (5% normal goat serum, 0.1% Triton X-100, and 0.05% Tween 20 in PBS) for 60 minutes. Sections were then incubated with AT8 (tau Ser202/Thr205), paired helical filament 1 (PHF1; tau Ser396/Ser404), R134d (total tau), 43D (tau 6–18), and 4G8 (amyloid precursor protein [APP]/Aβ) antibodies at 4 °C overnight. After washing three times for 15 minutes each with 10 mM PBS, sections were incubated with Alexa Fluor 488-conjugated goat antimouse or antirabbit IgG secondary antibodies (1:500; Molecular Probes, Eugene, OR, USA) in 10 mM PBS with 0.05% Tween 20 for 2 h at room temperature. Sections were subsequently washed, mounted, and coverslipped using ProLong Gold Antifade reagent with 4′,6-diamidino-2-phenylindole (DAPI; Thermo Fisher Scientific). All images were acquired using a Nikon Eclipse Ti microscope (Nikon Instruments, Melville, NY, USA) and MetaMorph microscopy automation and image analysis software (Molecular Devices, Sunnyvale, CA, USA), and ImageJ software (National Institutes of Health, Bethesda, MD, USA) was used to perform background subtraction and thresholding.

### Thioflavin S staining

Briefly, free-floating sagittal sections were rinsed in PBS for 10 minutes, then mounted onto glass slides and allowed to air-dry overnight. Then thioflavin S staining was performed as described before [28].

### Human Aβ_40_ and Aβ_42_ measurements by ELISA

The tissue from forebrain was homogenized in 10 volumes of ice-cold guanidine hydrochloride buffer (50 mM Tris-HCl, pH 8.0, 5.0 M guanidine HCl). The homogenate was mixed for 4 h at room temperature and then stored at −20 °C. For ELISA, each brain homogenate was diluted 1:25 with ice-cold reaction buffer (5% bovine serum albumin, 0.03% Tween 20, 2.1 mM AEBSF, 20 μg/ml aprotinin, 20 μg/ml leupeptin, 2.0 mM ethylenediaminetetraacetic acid, pH 7.4) in modified Dulbecco’s PBS (8 mM sodium phosphate, 2 mM potassium phosphate, 0.14 M NaCl, 10 mM KCl) and centrifuged at 16,000 × *g* for 20 minutes at 4 °C. The final concentration of AEBSF was 1 mM to prevent proteolysis of Aβ peptides, and the final concentration of guanidine hydrochloride was 0.1 M. The supernatant was further diluted 1:1 (vol/vol) with standard diluent buffer and assessed using an ELISA kit specific for human Aβ_40_ or Aβ_42_ and calibrated with synthetic Aβ peptides (catalogue number KHB3482; Invitrogen) according to the manufacturer’s instructions. The Aβ_40_ and Aβ_42_ peptide standards were prepared with the same composition of the buffer used for the dilution of the samples.

### Statistical analysis

Data were analyzed using Prism version 5.0 software (GraphPad Software Inc., La Jolla, CA, USA) and one-way or two-way analysis of variance (ANOVA) (as appropriate) followed by a Bonferroni post hoc test. Further intergroup comparisons were also performed using unpaired, two-tailed *t* tests. All data are presented as mean ± SEM. *p* < 0.05 was considered statistically significant.

## Results

### Passive immunization with tau antibodies rescues learning and memory deficits

**Fig. 2:**
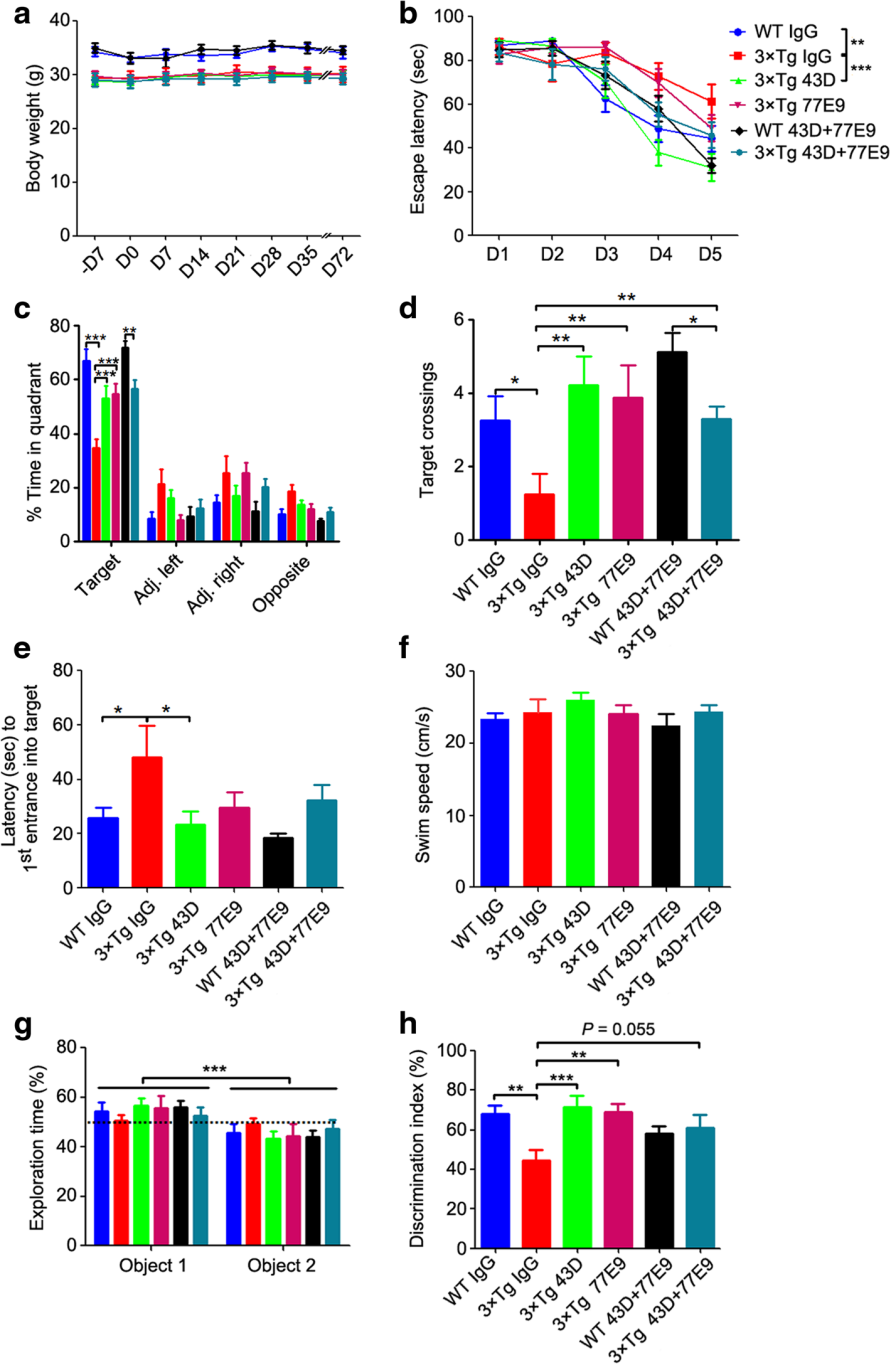
Immunization with tau antibodies 43D and 77E9 rescues cognitive impairment without any side effects in 3×Tg-AD mice. **a** The body weights of the mice were measured once per week. **b–f** The Morris water maze test was carried out after the sixth immunization (see Fig. 1). **b** The escape latency (in seconds) to reach the hidden platform during acquisition phase for 5 days (two-way ANOVA, WT-IgG vs. 3×Tg-IgG, *F* = 8.47, *dfd* = 90, *p* < 0.01; 3×Tg-IgG vs. 3×Tg-43D, *F* = 12.95, *dfd* = 75, *p* < 0.001; 3×Tg-IgG vs. 3×Tg-77E9, *F* = 0.23, *dfd* = 70, *p* = 0.63). **c** Percentage of time in the quadrant during the probe trial (two-way ANOVA, *F* = 241.59, *dfd* = 200, *p* < 0.0001; Bonferroni posttests for target quadrant, WT-IgG vs. 3×Tg-IgG, *p* < 0.01; 3×Tg-IgG vs. 3×Tg-43D, *p* < 0.001; 3×Tg-IgG vs. 3×Tg-77E9, *p* < 0.01). **d** Number of target crossings in the probe trial (one-way ANOVA, *F* = 3.666, *p* = 0.0067; Bonferroni multiple comparisons test, WT-IgG vs. 3×Tg-IgG, *p* < 0.05; 3×Tg-IgG vs. 3×Tg-43D, *p* < 0.01; 3×Tg-IgG vs. 3×Tg-77E9, *p* < 0.01). **e** Latency to first entrance into target zone (one-way ANOVA, *F* = 2.717, *p* = 0.0301; Bonferroni multiple comparisons test, WT-IgG vs. 3×Tg-IgG, *p* < 0.05; 3×Tg-IgG vs. 3×Tg-43D, *p* < 0.05). **f** The average swim speed during the probe trial (one-way ANOVA, *F* = 1.003, *p* = 0.4257). **g** and **h**, One-trial novel object recognition test (see Fig. 1). **g** The percentage of time spent exploring two identical objects during sample phase (two-way ANOVA, *F* = 18.93, *dfd* = 122, *p* < 0.0001). **h** Discrimination index (time spent exploring novel object/time spent exploring novel and familiar objects) × 100% in test phase (one-way ANOVA, *F* = 4.517, *p* = 0.0014; Bonferroni multiple comparisons test, WT-IgG vs. 3×Tg-IgG, *p* < 0.01; 3×Tg-IgG vs. 3×Tg-43D, *p* < 0.001; 3×Tg-IgG vs. 3×Tg-77E9, *p* < 0.01). *n* = 12 for WT mice treated with IgG or 43D + 77E9, 3×Tg-AD mice treated with 43D, 77E9, or 43D + 77E9; and *n* = 14 for 3×Tg-AD mice treated with IgG. Data are reported as mean ± SEM. *p < 0.05, **p < 0.01, ***p < 0.001 by ANOVA followed by a Bonferroni post hoc test. *AD* Alzheimer’s disease, *ANOVA* Analysis of variance, *IgG* Immunoglobulin G, 3×*Tg* Triple-transgenic, *WT* Wild type

To investigate whether passive immunization with tau antibodies 43D and 77E9 can rescue cognitive impairment in 3×Tg-AD mice, the Morris water maze task was conducted 1 day after the sixth immunization (Fig. 1). Passive immunization with 43D and 77E9 antibody neither caused any significant change in body weight (Fig. 2a) nor produced any neurological deficits during the entire period of this study. 3×Tg-AD mice immunized with 43D antibody took significantly less time than the mice treated with mouse IgG to find the hidden platform in the acquisition phase (Fig. 2b). More importantly, in the probe trial, 3×Tg-AD mice treated with 43D antibody spent longer in the target quadrant (Fig. 2c), had more target crossings (Fig. 2d), and took much less time to enter the target quadrant (Fig. 2e) than IgG-treated animals.

Unlike the mice immunized with 43D, the 77E9 antibody-immunized animals took amounts of time to reach the hidden platform in acquisition phase similar to those of the mouse IgG-treated controls (Fig. 2b). However, the mice treated with 77E9 showed similar performance as the 3×Tg-AD mice treated with 43D antibody in the probe trial (Fig. 2c–e). Animals immunized with a 1:1 mixture of 43D and 77E9 showed performance in the probe test similar to those treated with 43D alone. No significant difference was observed in swim speed during probe trials among all groups of animals (Fig. 2f). These results indicated that passive immunization targeting N-terminal tau, especially with 43D, could reverse spatial memory impairment without causing any side effects in 3×Tg-AD mice.

To examine whether immunization with tau antibodies could rescue the short-term memory impairment in 3×Tg-AD mice, we conducted a one-trial novel object recognition task with a 20-minute interval between the sample phase and the test phase. In the one-trial novel object recognition test, two identical objects, object 1 on location 1 and object 2 on location 2, were placed. For some unknown reason, the mice spent unequal amounts of time exploring the two identical objects during the 8-minute sample phase (Fig. 2g), which did not correlate with any of the two objects. Therefore, the less-explored object during sample phase was changed to a novel object during a 5-minute test phase. 3×Tg-AD mice spent less time exploring the novel object than the familiar object in the test phase than the WT mice did (Fig. 2h), whereas 3×Tg-AD mice immunized with 43D and 77E9 spent longer times exploring the novel object than did the 3×Tg-AD mice treated with mouse IgG, and amounts of time similar to WT mice (Fig. 2h). These results clearly indicate short-term memory impairment in 3×Tg-AD mice and its rescue by immunization with six doses of 43D and 77E9 antibodies.

### The beneficial effect of immunization with tau antibodies on cognition is sustained even after discontinuing immunization

**Fig. 3:**
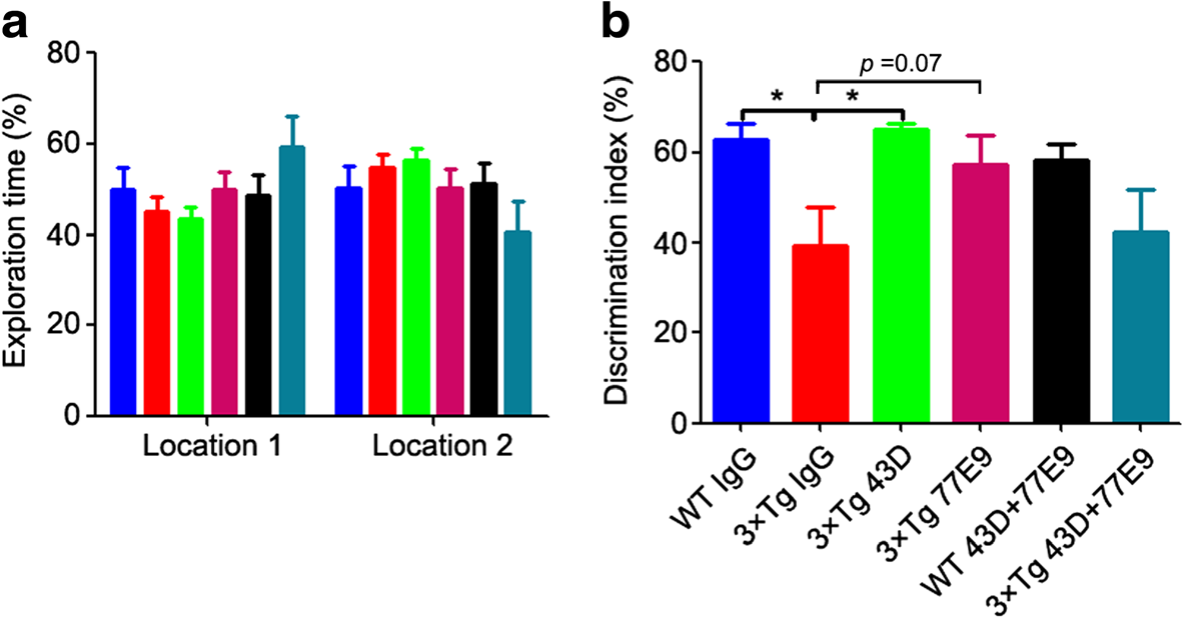
Immunization with tau antibodies 43D and 77E9 improves spatial memory beyond the period of immunization in 3×Tg-AD mice. Novel object location test was carried out 100 days after the last injection (see Fig. 1). **a** The percentage of time spent exploring two identical objects during sample phase (two-way ANOVA, *F* = 0.21, *dfd* = 56, *p* = 0.6484). **b** Discrimination index (time exploring object at novel location/time exploring object novel and familiar locations) × 100% in test phase (one-way ANOVA, *F* = 2.836, *p* = 0.0357; Bonferroni multiple comparisons test, WT-IgG vs. 3×Tg-IgG, *p* < 0.05; 3×Tg-IgG vs. 3×Tg-43D, *p* < 0.05). *n* = 6 and *n* = 5 for WT mice treated with IgG or 43D + 77E9, respectively; *n* = 6 for 3×Tg-AD mice treated with 43D, 77E9, or 43D + 77E9; and *n* = 8 for 3×Tg-AD mice treated with IgG. Data are reported as mean ± SEM. *p < 0.05 by one-way ANOVA followed by a Bonferroni post hoc test. *ANOVA* Analysis of variance, *IgG* Immunoglobulin G, 3×*Tg* Triple-transgenic, *WT* Wild type

To assess whether the beneficial effect of immunizations with 43D and 77E9 antibodies could last beyond the immunization period, the novel object location task was employed to measure the short-term memory at 100 days after the last immunization. All mice spent similar amounts of time exploring the objects at location 1 and location 2 in the sample phase (Fig. 3a). 3×Tg-AD mice immunized with 43D antibody spent much more time exploring the object in a new location than the mice treated with mouse IgG in the test phase (Fig. 3b). 3×Tg-AD mice immunized with 77E9 antibody also showed a clear trend (unpaired *t* test, 3×Tg-IgG vs. 3×Tg-77E9, *p* = 0.07) toward spending more time exploring the object at a novel location than did the mouse IgG-treated control mice (Fig. 3b). These results indicate that the beneficial effect of immunization with 43D and 77E9 antibodies on short-term memory can last at least approximately 3 months after discontinuing immunization.

### Two doses of immunization with 43D and 77E9 antibodies decreases total tau in the hippocampus

**Fig. 4:**
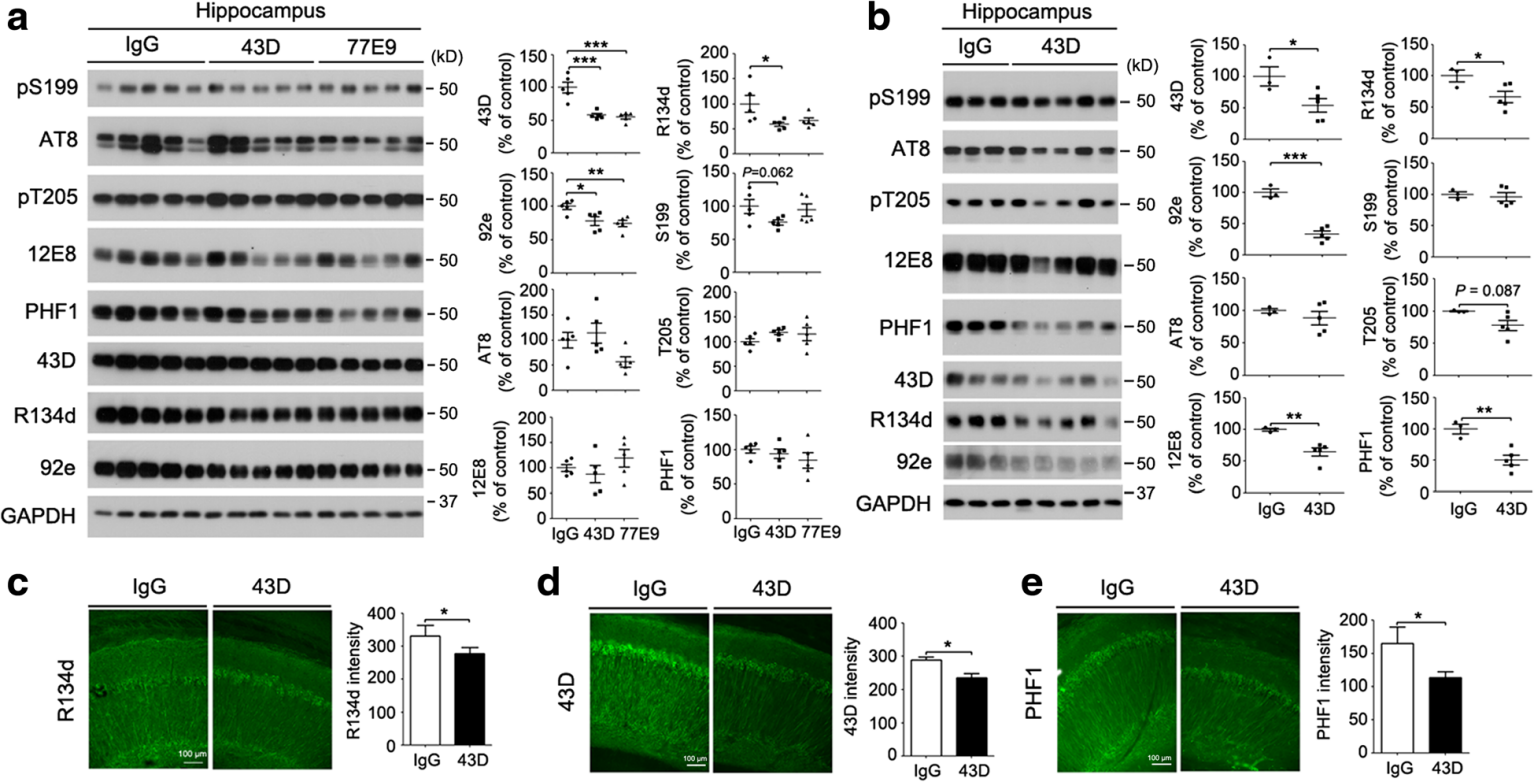
Immunization with 43D dose-dependently decreases tau pathology. Immunization with (**a**) two doses of 43D and 77E9 antibody or (**b–e**) six doses of 43D. Mice were killed 1 day after their last injection. **a** and **b** Representative Western blots of hippocampus developed with R134d and 92e against total tau, 43D against human transgenic tau, and several phosphorylation-dependent and site-specific tau antibodies. Densitometric quantification of blots after normalization with GAPDH is shown. Five of the 3×Tg-AD mice per group in (**a**) and three or five of the 3×Tg-AD mice for IgG or 43D treatment, respectively, are shown. Data are percentages of mouse IgG (100%)-treated animals reported as mean ± SEM. Immunostaining of CA1 from 3×Tg-AD mice immunized with six doses of 43D or control IgG with (**c**) R134d, (**d**) 43D, or (**e**) PHF1 antibodies. The quantification of R134d, 43D, and PHF1 immunostaining intensity in total CA1 region was based on two sections from each of three or five mice per group. Data are shown as mean ± SEM. *p < 0.05, **p < 0.01, ***p < 0.001 by ANOVA followed by a Bonferroni post hoc test for two doses of immunization or by unpaired two-tailed *t* test for six doses of immunization. *AD* Alzheimer’s disease, *ANOVA* Analysis of variance, *GAPDH* Glyceraldehyde 3-phosphate dehydrogenase, *IgG* Immunoglobulin G, 3×*Tg* Triple-transgenic

To investigate whether immunization with tau antibodies can reduce tau pathology in a dose-dependent manner, we first immunized the 12-month-old female 3×Tg-AD mice with mouse IgG, 43D, and 77E9 once per week for 2 weeks (15 μg/mouse intravenously), and then killed the mice 1 day after their second immunization. Both 43D and 77E9 reduced human transgenic tau (43D) and total tau level (tested with R134d and 92e antibodies) in the hippocampus (Fig. 4a). However, we did not observe a significant decrease of hyperphosphorylated tau after two doses of immunization with 43D or 77E9 antibodies (Fig. 4a). These results indicate that short-term immunization with normal N-terminal tau 43D and 77E9 antibodies can reduce total tau but not hyperphosphorylated tau levels in mouse brain.

### Six doses of immunization with 43D reduces both total and hyperphosphorylated tau in hippocampus

To determine the effect of long-term immunization, we immunized 15-month-old 3×Tg-AD mice with mouse IgG and 43D once weekly for 6 weeks (15 μg/mouse intravenously), and we killed the mice 1 day after their last immunization. Similarly to two doses, six doses of immunization decreased both human transgenic tau assayed with antibody 43D and total tau level determined with antibodies R134d and 92e in hippocampus (Fig. 4b). Importantly, six doses also significantly reduced hyperphosphorylated tau at Ser262/Ser356 (12E8) and Ser396/Ser404 (PHF1) sites, and it also showed a clear trend toward reducing tau hyperphosphorylation at Thr205 in hippocampus (Fig. 4b–e). These results indicate that long-term immunization with normal N-terminal tau antibody 43D could decrease tau pathology in mouse brain.

### Immunization with 43D but not 77E9 decreases APP and Aβ accumulation in moderate to severe stages of plaque pathology

**Fig. 5:**
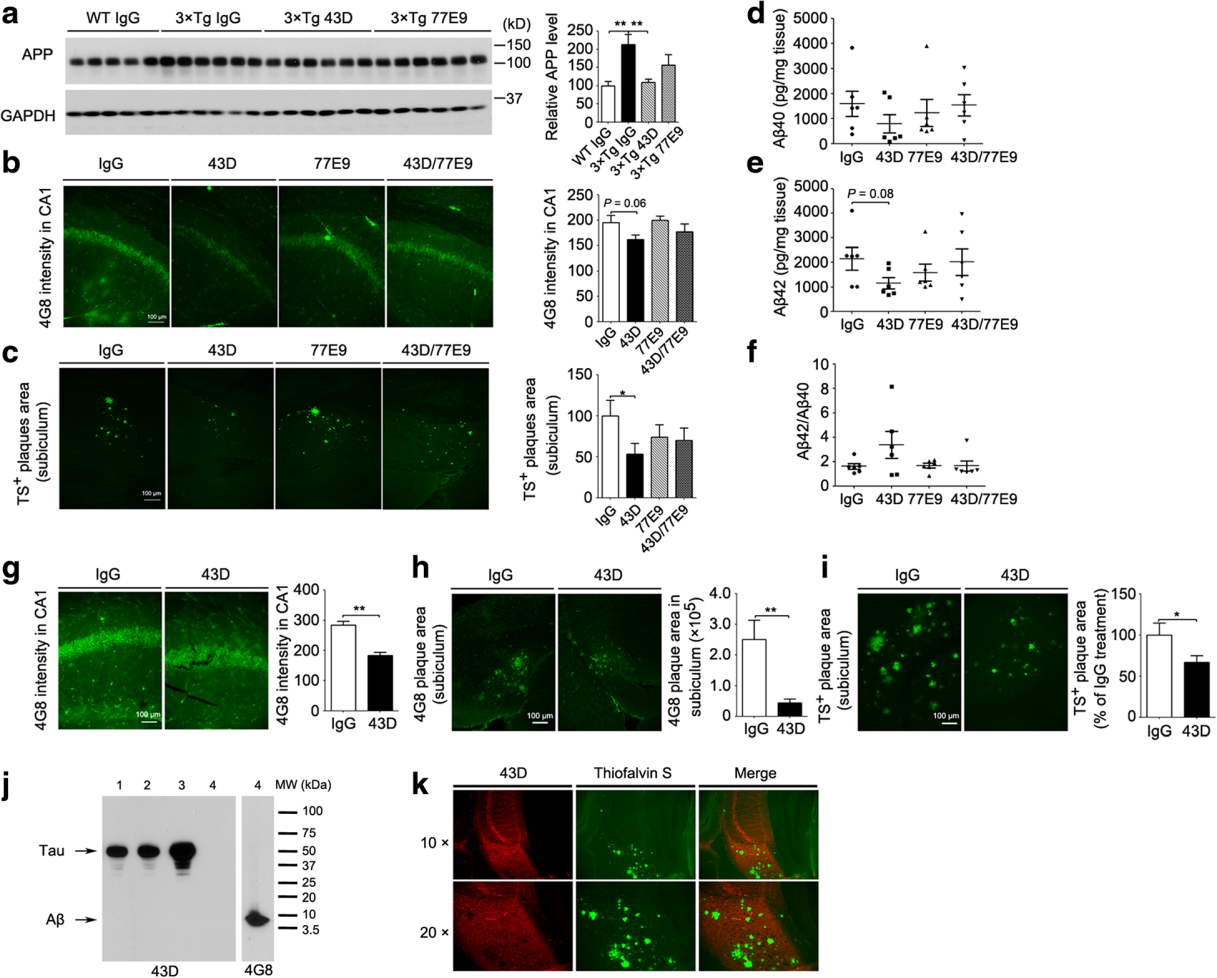
Immunization with 43D but not 77E9 decreases Aβ pathology in 3×Tg-AD mice. **a**–**f** Analysis of Aβ pathology 1 week after seventh dose (see Fig. 1). **a** Representative Western blots of forebrain developed with APP. Densitometric quantification of blots after normalization with GAPDH. Immunostaining of (**b**) CA1 with 4G8 and (**c**) subiculum with thioflavin S in 3×Tg-AD mice. Quantification of APP staining with 4G8 in CA1 and amyloid plaques in subiculum stained with thioflavin S from two sections per mouse of five or six mice per group. The levels of total (**d**) Aβ_40_ and (**e**) Aβ_42_ (unpaired *t* test, 3×Tg-IgG vs. 3×Tg-43D, *p* = 0.08) and (**f**) the ratio of Aβ_42_/Aβ_40_ in forebrain was quantified by ELISA. **g–i** Aβ pathology in 3×Tg-AD mice 1 day after the sixth injection. 4G8 staining in (**g**) CA1 and (**h**) subiculum and (**i**) thioflavin S staining in subiculum in 3×Tg-AD mice. 43D did not cross-react with Aβ (**j**) and Aβ plaques (**k**). **j** Thirty micrograms of 3×Tg-AD mouse brain homogenate (*lanes 1–3*) and 0.4 μg of purified Aβ (*lane 4*) were loaded. Nitrocellulose membrane was first developed with 43D antibody (*left panel*) and then developed with 4G8 antibody (*right panel*). **k** Double-stained 3×Tg-AD mouse brain sections with 43D antibody and thioflavin S. *Top row*: CA1 and subiculum area (original magnification × 10). *Bottom row*: Subiculum area (original magnification × 20). Quantification of APP staining in CA1 and amyloid plaques in subiculum stained with 4G8 and thioflavin S from two sections per mouse of three or five animals per group. Data are shown as mean ± SEM. *p < 0.05, **p < 0.01, ***p < 0.001 by ANOVA followed by a Bonferroni post hoc test for two doses of immunization or by unpaired two-tailed *t* test for six doses of immunization. *AD* Alzheimer’s disease, *ANOVA* Analysis of variance, *APP* Amyloid precursor protein, *Aβ* Amyloid-β, *ELISA* Enzyme-linked immunosorbent assay, *GAPDH* Glyceraldehyde 3-phosphate dehydrogenase, *IgG* Immunoglobulin G, *MW* Molecular weight, 3×*Tg* Triple-transgenic, *TS* Thioflavin S, *WT* Wild type

3×Tg-AD mice develop amyloid plaques starting around 9 months of age. They are first apparent in the cortex and progress to the hippocampus with age [42–44]. In the present study, we determined whether immunization with tau antibodies could alter the level of APP and Aβ plaques. Immunization with 43D but not 77E9 antibody decreased APP levels in forebrain (Fig. 5a) and showed a trend of reduced APP staining in CA1 (Fig. 5b). Immunization with 43D also showed a reduction in thioflavin S-positive Aβ plaque load in subiculum (Fig. 5c) and showed a trend toward reduction in the levels of Aβ_40_ and Aβ_42_ (unpaired *t* test, 3×Tg-IgG vs. 3×Tg-43D, *p* = 0.08) in forebrain (Fig. 5d–f) at 6 days after the last dose. However, the levels of Aβ_40_ and Aβ_42_, as well as the ratio of Aβ_42_/Aβ_40_, were similar among 43D, 77E9, and mouse IgG-immunized 3×Tg-AD mice at 16 weeks after the last dose (data not shown).

Consistently, immunization with 43D decreased APP levels and Aβ plaque loads 1 day after the sixth immunization (Fig. 5g–i). Importantly, there was no cross-reaction between 43D and Aβ or Aβ plaques (Fig. 5j and k). These data suggest that immunotherapy targeting tau 6–18 but not tau 184–195 could reduce the amyloid plaque load, which can persist long after the discontinuation of the treatment.

### Immunization with 43D leads to activation of microglia and increased levels of complement components C1q and C9

**Fig. 6:**
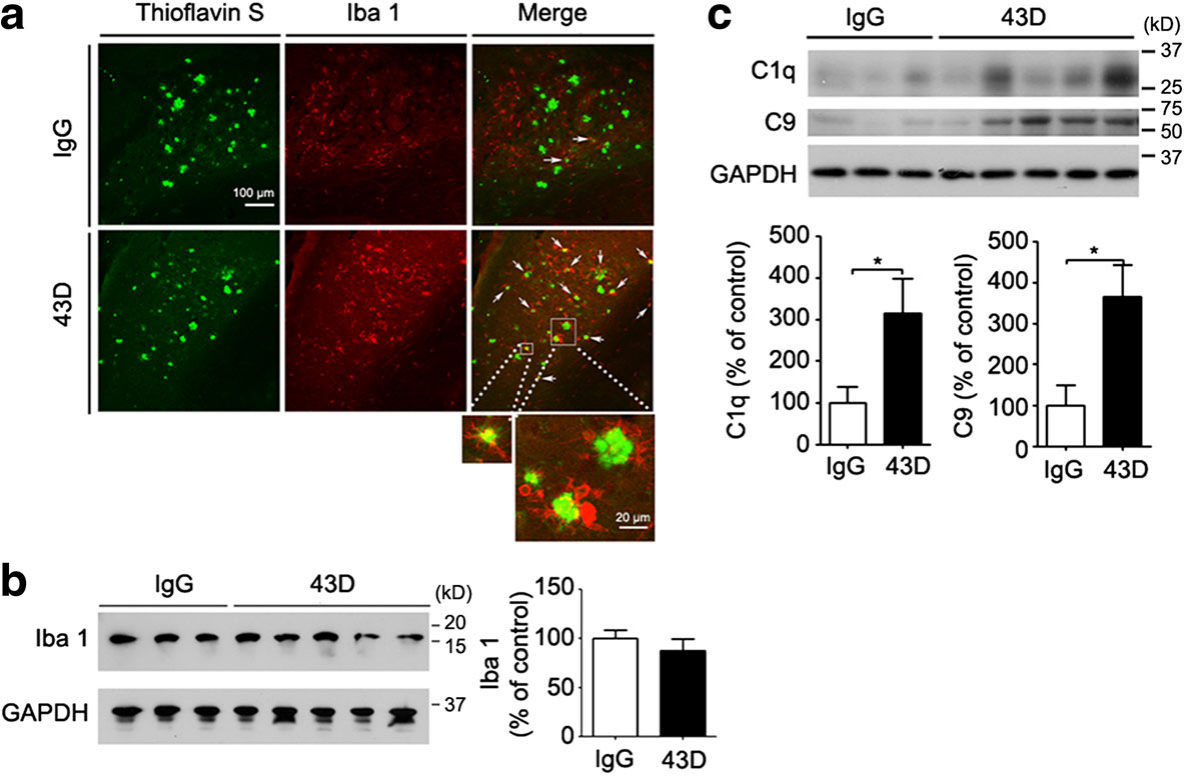
Immunization with 43D increases the activity of microglia and the complement system in 3×Tg-AD mice. The activation of microglia and the complement system was assessed 1 day after the sixth injection. **a** Thioflavin S staining and Iba1 immunofluorescence were performed in the subiculum area. *White arrows* highlight activated microglial cells aggregated around Aβ plaques. Representative Western blots of hippocampus developed (**b**) with Iba1 antibody and (**c**) with complement components C1q and C9 antibodies and densitometric quantification of blots after normalization with GAPDH. Three or five representative 3×Tg-AD mice in the IgG or 43D treatment group, respectively, are shown. Data are percentages of mouse IgG (100%)-treated animals, reported as mean ± SEM. *p < 0.05 by unpaired two-tailed Student’s *t* test. *AD* Alzheimer’s disease, *Aβ* Amyloid-β, *GAPDH* Glyceraldehyde 3-phosphate dehydrogenase, *IgG* Immunoglobulin G, 3×*Tg* Triple-transgenic

To explore the possible mechanisms involved by which immunization with 43D ameliorates Aβ pathology, first we investigated the involvement of microglia. Although the microglial level tested by Western blots with Iba1 antibody did not show a significant difference between 43D- and IgG-treated mice in the hippocampus (Fig. 6b), the 43D-immunized mice showed much more activated microglia with larger somata and short, thick extensions than the mice treated with mouse IgG in the subiculum region (Fig. 6a). Importantly, activated microglia were found to be aggregated around thioflavin S-positive plaques in phagocytizing positions (Fig. 6a). These data suggest that 43D immunization with 43D draws microglia to Aβ plaques. Additionally, immunization with 43D also increased C1q and C9 levels (Fig. 6c), which suggests that this treatment could possibly induce the activation of complement system and consequently the clearance of Aβ plaques by microglia in 3×Tg-AD mice.

### Tau hyperphosphorylation is reduced beyond the period of immunization with 43D and 77E9

**Fig. 7:**
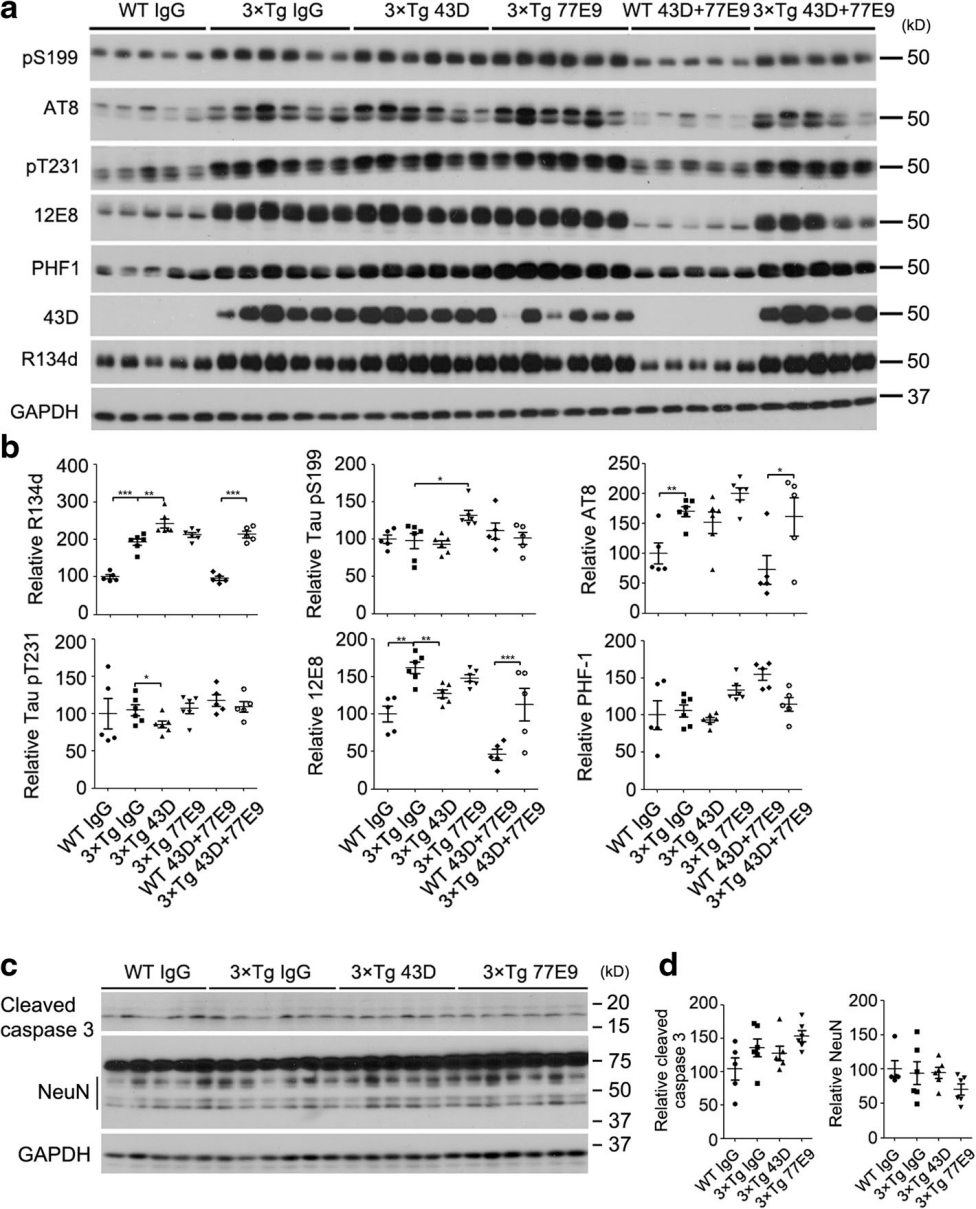
Effect of immunization with 43D and 77E9 antibodies on tau pathology lasts several weeks after the last treatment. **a** Representative Western blots of forebrain developed with R134d against total tau and with several phosphorylation-dependent and site-specific tau antibodies. **b** Densitometric quantification of phosphorylated tau after normalization with total tau (R134d). **c** Western blots of forebrain developed with antibodies to apoptotic marker cleaved caspase-3 and neuronal marker NeuN. **d** Quantification of cleaved caspase-3 and NeuN from (**c**). *n* = 5 for WT mice treated with IgG, 43D, and 77E9, and 3×Tg-AD mice treated with 43D + 77E9; *n* = 6 for 3×Tg-AD mice treated with IgG, 43D, and 77E9. Data are percentages of mouse IgG (100%)-treated animals, reported as mean ± SEM. *p < 0.05, **p < 0.01 by one-way ANOVA followed by Bonferroni post hoc test. *AD* Alzheimer’s disease, *ANOVA* Analysis of variance, *Aβ* Amyloid-β, *GAPDH* Glyceraldehyde 3-phosphate dehydrogenase, *IgG* Immunoglobulin G, *pS199* Phospho-tau (Ser199), *pT205* Phospho-tau (Thr205), 3×*Tg* Triple-transgenic, *WT* Wild type

To assess whether immunization with antibodies 43D and 77E9 reduces tau hyperphosphorylation beyond the period of the treatment, we investigated the levels of total and hyperphosphorylated taus by Western blot analysis in the forebrain 1 and 16 weeks after the last immunization. We found an increase of total tau but a decrease in tau hyperphosphorylation (p-tau normalized with total tau) at Thr231 and 12E8 (Ser262/Ser356) sites, as well as a trend at PHF1 (Ser396/Ser404, *p* = 0.098) sites in the forebrain of 43D-immunized 3×Tg-AD mice (Fig. 7a and b). Similar results were found in the forebrain at 16 weeks after the last injection (data not shown). These findings suggested that the beneficial effect of immunization with 43D could last beyond the period of the treatment.

Additionally, we did not observe any change of an apoptotic marker, cleaved caspase-3, and a neuronal marker, NeuN, in forebrain (Fig. 7c and d). These data indicate that treatments with IgG and tau antibodies 43D and 77E9 did not cause neuronal apoptosis or change the neuronal numbers.

## Discussion

A central objective of dementia field is to inhibit, or at least effectively modify, the course of AD. In the present study, we examined the dose-dependent effect of treatment with tau antibodies on pathology and cognitive performance and how long the beneficial effect could be sustained after discontinuing immunization. We found that two doses of immunization with tau antibodies (15 μg/mouse, intravenously, once per week) could decrease the total tau level, but not the level of hyperphosphorylated tau, in 3×Tg-AD mice. Six doses of immunization, however, reduced both total tau and hyperphosphorylated tau levels in hippocampus and rescued both spatial reference and episodic memory impairments in 3×Tg-AD mice. Importantly, the beneficial effect on short-term reference memory was sustained at least 3 months after discontinuation of immunization, as determined by a novel object location test. Moreover, immunization with 43D antibody induced activation of microglia and the complement system, which could have facilitated the clearance of Aβ plaques in 3×Tg-AD mice.

3×Tg-AD mice develop Aβ accumulation at approximately 9 months of age and tau pathology starting at 12 months of age [42–44]. Immunization of 12-month-old female 3×Tg-AD mice with 43D and 77E9 antibodies once weekly for 6 weeks rescued cognitive impairment, and the beneficial effect on cognitive improvement lasted at least 3 months after discontinuation of the treatment with tau antibodies. However, we observed a small change in tau pathology by Western blot analysis after behavioral tests that showed a marked improvement in cognitive performance. These findings suggest that the beneficial effect on behavioral improvement may last much longer than the effect on tau pathology, as well as that a certain threshold of the pathology is probably required to affect cognitive performance. The bulk of the tau pathology develops from about 12–18 months of age in 3×Tg-AD mice, and it is possible that the rate of tau pathology formation was more than its clearance after the discontinuance of the immunization. Therefore, to investigate whether tau immunization has a dose-dependent beneficial effect on tau pathology, we immunized the 3×Tg-AD mice employing two doses and six doses once per week (15 μg/mouse intravenously) and killed the mice 1 day later. We found that two doses of immunization decreased total tau but not hyperphosphorylated tau, whereas six doses of tau antibodies reduced both total tau and hyperphosphorylated tau. These data indicate that immunization with tau antibodies dose-dependently ameliorates the pathology in 3×Tg-AD mice and that extracellular tau, which is mostly nonhyperphosphorylated, is the primary target of the tau antibodies.

Tau pathology is well documented to propagate in a predictable pattern [37, 38]. Tau immunotherapy can target its transcellular propagation and clear pathological tau. Recently, several studies indicated that tau antibodies can be taken up by neurons and promote intracellular sequestration and clearance of tau [16, 20, 25, 54–57]. Fc receptor-mediated endocytosis and the endosome-autophagosome-lysosome system are believed to play a critical role in antibody-mediated clearance of tau pathology. In a neuron, antibodies localize in the endosomal-autophagosome-lysosome system and promote tau clearance by disassembling tau aggregates. Importantly, antibody uptake into neurons has been shown to be a prerequisite for acute tau clearance. This intracellular interaction may sequester the tau protein, preventing its release into the extracellular space and subsequent spread in the brain [58]. In the present study, 43D was found to recognize human tau in neuronal somata and dendrites (Fig. 4d) and 77E9 mostly labeled tau in axons (data not shown). Whether both mechanisms are involved in the beneficial effects of 43D and 77E9 immunization is not currently understood.

Tau is primarily a cytoplasmic protein that stabilizes microtubules. Recent studies have shown that tau can be released into the extracellular space from neurons as a physiological process that is independent from neuronal death [59, 60]. Additionally, an increase in the level of tau in cerebrospinal fluid (CSF) is associated with AD and is linked to Aβ deposition [61, 62]. Extracellular tau seeds can enter neurons and further induce intracellular tau accumulation and subsequent spreading of tau pathology. Importantly, mostly N-terminal but not C-terminal fragments are found in extracellular tau in AD CSF [63, 64]. In the present study, we found that two doses of immunization decreased only total tau, but that six doses removed both total and hyperphosphorylated tau. Two doses of immunization with 43D against N-terminal tau 6–18 probably first clears the extracellular tau, which is largely nonhyperphosphorylated [65]. However, a total of six doses of immunization with 43D and 77E9 antibodies probably removes both extracellular and intracellular tau.

Notably, we found that immunization with 43D decreased Aβ pathology, though 43D did not cross-react with Aβ and Aβ plaques. Our findings are consistent with studies that showed that tau pathology precedes Aβ pathology in aged and AD brains [66, 67]. One possible mechanism is that 43D treatment rescues neurofibrillary degeneration and decreases Aβ production via reduction of APP synthesis and/or its amyloidogenic processing, because immunization with 43D decreased both APP and Aβ levels in the forebrain. Clearance of extracellular tau with tau antibodies probably inhibits Aβ production through inhibition of neurofibrillary degeneration [63]. Therefore, immunotherapy targeting N-terminal tau not only directly decreases tau pathology but also ameliorates Aβ pathology, which may form a positive feedforward regulation in vivo. The exact nature of the molecular mechanism involved in the reduction of APP and Aβ production by tau immunization is not currently understood. We speculate that the reduction in amyloidogenic processing of APP in tau-immunized mice could be secondary to improvement in neuronal connectivity as a result of decreased neurofibrillary degeneration.

One prominent feature of AD neuropathology is the association of activated proteins of the classical complement pathway with the lesions. The full range of classical pathway complement proteins from C1q to C5b-9 (membrane attack complex) has been demonstrated by immunocytochemical staining to be associated with senile plaques, NFTs, neuropil threads, and dystrophic neurites in AD brain [68, 69]. Binding of C1q and C3 to amyloid plaques can activate the classical and alternative pathways of the complement system. The complement system may be useful in eliminating aggregated and toxic proteins associated with these lesions and thus may have a protective effect [70]. C3 plays a detrimental role in complement full activation. Knockout of C3 or overexpression of C3 convertase inhibitor (Crry) was reported to increase the amyloid load burden but to reduce phagocytic microglia [71, 72]. Microglia are the phagocytes of the brain and express complement receptor CR3. C3 promotes plaque clearance via triggering of Aβ phagocytosis by microglia [73, 74]. Although in the present study the C3 level was undetectable by Western blotting in brains of 3×Tg-AD mice, immunization with 43D increased the levels of C1q, the first protein in the complement cascade, and C9, the late-stage activation marker, and it promoted microglial activation and aggregation around Aβ plaques. We did not observe an association between C1q and Aβ plaques by immunohistochemistry in 3×Tg-AD mice, which is consistent with a previous report [75]. Currently, the exact mechanism is not known. We suspect that inhibition of Aβ pathology occurs by passive immunization with 43D, probably on one hand owing to improvement of neuronal connectivity leading to a decrease in amyloidogenic processing of APP and on the other hand owing to enhancement of the clearance of amyloid plaque load by activation of the complement and phagocytosis by the microglia.

Recently, tau immunotherapy has shown promising beneficial effects in animal models, and several clinical trials on tau immunotherapy are underway or are at various discovery stages [58]. The key question in tau immunotherapy is which site/domain to target. D’Abramo et al. demonstrated that MC1 (tau 312–322) targeting PHF-tau was more efficacious than the high-affinity DA31 (tau 150–190) antibody against normal tau, which suggests that antibody specificity is more important than its affinity in therapeutic applications [76]. Tau antibodies HJ8.5 (tau 25–30), HJ 9.4 (tau 7–13), and HJ9.3 (tau 306–320) were developed by blocking tau seeding in vitro, but only HJ8.5 and HJ9.4, and not HJ9.3, rescued contextual fear deficits, though HJ8.5 and HJ9.3 strongly decreased pathological tau seeds in vivo [23]. In the present study, 43D and 77E9 targeted tau 6–18 and tau 184–195, respectively, and the isotype of both antibodies is IgG1 kappa light chain. Thus, the different domains of tau-targeted epitope by 43D and 77E9 may represent one possible explanation for better effects with 43D than with 77E9 in reducing tau pathology, rescuing cognitive deficits, and ameliorating the Aβ pathology. These data imply that the sites/domains targeted by tau antibodies may play a pivotal role in the beneficial therapeutic outcome.

## Conclusions

The present study shows that passive immunization targeting N-terminal tau 6–18 and 184–195 with 43D and 77E9 antibodies can reduce total and hyperphosphorylated tau levels dose-dependently and can rescue spatial and short-term episodic memory impairments. Importantly, the beneficial effect of immunization with 43D and 77E9 on tau hyperphosphorylation and short-term memory improvement is sustained at least 3 months after discontinuation of the immunization. The passive immunization with tau antibody 43D can also decrease APP, amyloid plaque load, and Aβ level. Overall, this study shows that passive immunization targeting the N-terminal projection domain of tau offers a potential promising treatment opportunity for AD and probably other tauopathies.

## Abbreviations

AD: Alzheimer’s disease
AEBSF: 4-(2-Aminoethyl)benzenesulfonyl fluoride hydrochloride
ANOVA: Analysis of variance
APP: Amyloid precursor protein
Aβ: Amyloid-β
CSF: Cerebrospinal fluid
ELISA: Enzyme-linked immunosorbent assay
GAPDH: Glyceraldehyde 3-phosphate dehydrogenase
IgG: Immunoglobulin G
IHC: Immunohistochemistry
MWM: Morris water maze
NFT: Neurofibrillary tangle
NOR: Novel object recognition
PHF: Paired helical filament
pS199: Phospho-tau (Ser199)
pT205: Phospho-tau (Thr205)
p-tau: Phosphorylated tau
3×Tg: Triple-transgenic
TS: Thioflavin S
WT: Wild type

## References

[1] Alzheimer’s Association (2015). 2015 Alzheimer’s disease facts and figures. Alzheimers Dement..

[2] Glenner GG, Wong CW (1984). Alzheimer’s disease: initial report of the purification and characterization of a novel cerebrovascular amyloid protein. Biochem Biophys Res Commun..

[3] Grundke-Iqbal I, Iqbal K, Tung YC, Quinlan M, Wisniewski HM, Binder LI (1986). Abnormal phosphorylation of the microtubule-associated protein tau (tau) in Alzheimer cytoskeletal pathology. Proc Natl Acad Sci U S A..

[4] Hardy J, Selkoe DJ (2002). The amyloid hypothesis of Alzheimer’s disease: progress and problems on the road to therapeutics. Science..

[5] Giacobini E, Gold G (2013). Alzheimer disease therapy—moving from amyloid-β to tau. Nat Rev Neurol..

[6] Reitz C (2012). Alzheimer’s disease and the amyloid cascade hypothesis: a critical review. Int J Alzheimers Dis..

[7] Doody RS, Thomas RG, Farlow M, Iwatsubo T, Vellas B, Joffe S (2014). Phase 3 trials of solanezumab for mild-to-moderate Alzheimer’s disease. N Engl J Med..

[8] Salloway S, Sperling R, Fox NC, Blennow K, Klunk W, Raskind M (2014). Two phase 3 trials of bapineuzumab in mild-to-moderate Alzheimer’s disease. N Engl J Med..

[9] Vandenberghe R, Rinne JO, Boada M, Katayama S, Scheltens P, Vellas B (2016). Bapineuzumab for mild to moderate Alzheimer’s disease in two global, randomized, phase 3 trials. Alzheimers Res Ther.

[10] Pasquier F, Sadowsky C, Holstein A, Leterme Gle P, Peng Y, Jackson N (2016). Two phase 2 multiple ascending-dose studies of vanutide cridificar (ACC-001) and QS-21 adjuvant in mild-to-moderate Alzheimer’s disease. J Alzheimers Dis..

[11] Alafuzoff I, Iqbal K, Friden H, Adolfsson R, Winblad B (1987). Histopathological criteria for progressive dementia disorders: clinical-pathological correlation and classification by multivariate data analysis. Acta Neuropathol..

[12] Arriagada PV, Growdon JH, Hedley-Whyte ET, Hyman BT (1992). Neurofibrillary tangles but not senile plaques parallel duration and severity of Alzheimer’s disease. Neurology..

[13] Terry RD, Masliah E, Salmon DP, Butters N, DeTeresa R, Hill R (1991). Physical basis of cognitive alterations in Alzheimer’s disease: synapse loss is the major correlate of cognitive impairment. Ann Neurol..

[14] Giannakopoulos P, Herrmann FR, Bussière T, Bouras C, Kövari E, Perl DP (2003). Tangle and neuron numbers, but not amyloid load, predict cognitive status in Alzheimer’s disease. Neurology..

[15] Sigurdsson EM (2016). Tau immunotherapy. Neurodegener Dis..

[16] Asuni AA, Boutajangout A, Quartermain D, Sigurdsson EM (2007). Immunotherapy targeting pathological tau conformers in a tangle mouse model reduces brain pathology with associated functional improvements. J Neurosci..

[17] Bi M, Ittner A, Ke YD, Gotz J, Ittner LM (2011). Tau-targeted immunization impedes progression of neurofibrillary histopathology in aged P301L tau transgenic mice. PLoS One..

[18] Boimel M, Grigoriadis N, Lourbopoulos A, Haber E, Abramsky O, Rosenmann H (2010). Efficacy and safety of immunization with phosphorylated tau against neurofibrillary tangles in mice. Exp Neurol..

[19] Boutajangout A, Ingadottir J, Davies P, Sigurdsson EM (2011). Passive immunization targeting pathological phospho-tau protein in a mouse model reduces functional decline and clears tau aggregates from the brain. J Neurochem..

[20] Gu J, Congdon EE, Sigurdsson EM (2013). Two novel Tau antibodies targeting the 396/404 region are primarily taken up by neurons and reduce Tau protein pathology. J Biol Chem..

[21] Troquier L, Caillierez R, Burnouf S, Fernandez-Gomez FJ, Grosjean ME, Zommer N (2012). Targeting phospho-Ser422 by active tau immunotherapy in the THYTau22 mouse model: a suitable therapeutic approach. Curr Alzheimer Res..

[22] Theunis C, Crespo-Biel N, Gafner V, Pihlgren M, Lopez-Deber MP, Reis P, et al. Efficacy and safety of a liposome-based vaccine against protein Tau, assessed in tau.P301L mice that model tauopathy. PLoS One. 2013;8:e72301. 10.1371/journal.pone.0072301 PMC3747157 23977276

[23] Yanamandra K, Kfoury N, Jiang H, Mahan TE, Ma S, Maloney SE (2013). Anti-tau antibodies that block tau aggregate seeding in vitro markedly decrease pathology and improve cognition in vivo. Neuron..

[24] Chai X, Wu S, Murray TK, Kinley R, Cella CV, Sims H (2011). Passive immunization with anti-Tau antibodies in two transgenic models: reduction of Tau pathology and delay of disease progression. J Biol Chem..

[25] Collin L, Bohrmann B, Gopfert U, Oroszlan-Szovik K, Ozmen L, Gruninger F (2014). Neuronal uptake of tau/pS422 antibody and reduced progression of tau pathology in a mouse model of Alzheimer’s disease. Brain..

[26] Sankaranarayanan S, Barten DM, Vana L, Devidze N, Yang L, Cadelina G (2015). Passive immunization with phospho-tau antibodies reduces tau pathology and functional deficits in two distinct mouse tauopathy models. PLoS One..

[27] Umeda T, Eguchi H, Kunori Y, Matsumoto Y, Taniguchi T, Mori H (2015). Passive immunotherapy of tauopathy targeting pSer413-tau: a pilot study in mice. Ann Clin Transl Neurol..

[28] Dai CL, Chen X, Kazim SF, Liu F, Gong CX, Iqbal K (2015). Passive immunization targeting the N-terminal projection domain of tau decreases tau pathology and improves cognition in a transgenic mouse model of Alzheimer disease and tauopathies. J Neural Transm (Vienna).

[29] Anand K, Sabbagh M (2015). Early investigational drugs targeting tau protein for the treatment of Alzheimer’s disease. Expert Opin Investig Drugs..

[30] Iqbal K, Grundke-Iqbal I, Zaidi T, Merz PA, Wen GY, Shaikh SS (1986). Defective brain microtubule assembly in Alzheimer’s disease. Lancet..

[31] Alonso AC, Zaidi T, Grundke-Iqbal I, Iqbal K (1994). Role of abnormally phosphorylated tau in the breakdown of microtubules in Alzheimer disease. Proc Natl Acad Sci U S A..

[32] Alonso AC, Grundke-Iqbal I, Iqbal K (1996). Alzheimer’s disease hyperphosphorylated tau sequesters normal tau into tangles of filaments and disassembles microtubules. Nat Med..

[33] Clavaguera F, Bolmont T, Crowther RA, Abramowski D, Frank S, Probst A (2009). Transmission and spreading of tauopathy in transgenic mouse brain. Nat Cell Biol..

[34] Clavaguera F, Akatsu H, Fraser G, Crowther RA, Frank S, Hench J (2013). Brain homogenates from human tauopathies induce tau inclusions in mouse brain. Proc Natl Acad Sci U S A..

[35] Sanders DW, Kaufman SK, DeVos SL, Sharma AM, Mirbaha H, Li A (2014). Distinct tau prion strains propagate in cells and mice and define different tauopathies. Neuron..

[36] Hu W, Zhang X, Tung YC, Liu F, Iqbal K (2016). Hyperphosphorylation determines both the spread and the morphology of tau pathology. Alzheimers Dement..

[37] Mohamed NV, Herrou T, Plouffe V, Piperno N, Leclerc N (2013). Spreading of tau pathology in Alzheimer’s disease by cell-to-cell transmission. Eur J Neurosci..

[38] Avila J, Simon D, Diaz-Hernandez M, Pintor J, Hernandez F (2014). Sources of extracellular tau and its signaling. J Alzheimers Dis..

[39] Flight MH (2013). Neurodegenerative disease: tau immunotherapy targets transcellular propagation. Nat Rev Drug Discov..

[40] Frost B, Jacks RL, Diamond MI (2009). Propagation of tau misfolding from the outside to the inside of a cell. J Biol Chem..

[41] Zilka N, Kontsekova E, Novak M (2008). Chaperone-like antibodies targeting misfolded tau protein: new vistas in the immunotherapy of neurodegenerative foldopathies. J Alzheimers Dis..

[42] Oddo S, Caccamo A, Shepherd JD, Murphy MP, Golde TE, Kayed R (2003). Triple-transgenic model of Alzheimer’s disease with plaques and tangles: intracellular Aβ and synaptic dysfunction. Neuron..

[43] Blanchard J, Wanka L, Tung YC, Del Carmen Cárdenas-Aguayo M, LaFerla FM, Iqbal K (2010). Pharmacologic reversal of neurogenic and neuroplastic abnormalities and cognitive impairments without affecting Aβ and tau pathologies in 3×Tg-AD mice. Acta Neuropathol..

[44] Kazim SF, Blanchard J, Dai CL, Tung YC, LaFerla FM, Iqbal K (2014). Disease modifying effect of chronic oral treatment with a neurotrophic peptidergic compound in a triple transgenic mouse model of Alzheimer’s disease. Neurobiol Dis..

[45] Kascsak RJ, Rubenstein R, Merz PA, Tonna-DeMasi M, Fersko R, Carp RI (1987). Mouse polyclonal and monoclonal antibody to scrapie-associated fibril proteins. J Virol..

[46] Morris RG, Garrud P, Rawlins JN, O’Keefe J (1982). Place navigation impaired in rats with hippocampal lesions. Nature..

[47] D’Hooge R, De Deyn PP (2001). Applications of the Morris water maze in the study of learning and memory. Brain Res Brain Res Rev..

[48] Vorhees CV, Williams MT (2006). Morris water maze: procedures for assessing spatial and related forms of learning and memory. Nat Protoc..

[49] Bevins RA, Besheer J (2006). Object recognition in rats and mice: a one-trial non-matching-to-sample learning task to study ‘recognition memory’. Nat Protoc..

[50] Antunes M, Biala G (2012). The novel object recognition memory: neurobiology, test procedure, and its modifications. Cogn Process..

[51] Contestabile A, Greco B, Ghezzi D, Tucci V, Benfenati F, Gasparini L (2013). Lithium rescues synaptic plasticity and memory in Down syndrome mice. J Clin Invest..

[52] Assini FL, Duzzioni M, Takahashi RN (2009). Object location memory in mice: pharmacological validation and further evidence of hippocampal CA1 participation. Behav Brain Res..

[53] Murai T, Okuda S, Tanaka T, Ohta H (2007). Characteristics of object location memory in mice: behavioral and pharmacological studies. Physiol Behav..

[54] Congdon EE, Gu J, Sait HB, Sigurdsson EM (2013). Antibody uptake into neurons occurs primarily via clathrin-dependent Fcγ receptor endocytosis and is a prerequisite for acute tau protein clearance. J Biol Chem..

[55] Krishnamurthy PK, Deng Y, Sigurdsson EM (2011). Mechanistic studies of antibody-mediated clearance of tau aggregates using an ex vivo brain slice model. Front Psychiatry..

[56] Watkinson RE, McEwan WA, James LC (2014). Intracellular antibody immunity. J Clin Immunol..

[57] Shamir DB, Rosenqvist N, Rasool S, Pedersen JT, Sigurdsson EM (2016). Internalization of tau antibody and pathological tau protein detected with a flow cytometry multiplexing approach. Alzheimers Dement..

[58] Pedersen JT, Sigurdsson EM (2015). Tau immunotherapy for Alzheimer’s disease. Trends Mol Med..

[59] Chai X, Dage JL, Citron M (2012). Constitutive secretion of tau protein by an unconventional mechanism. Neurobiol Dis..

[60] Shah KH, Zhang B, Ramachandran V, Herman PK (2013). Processing body and stress granule assembly occur by independent and differentially regulated pathways in *Saccharomyces cerevisiae*. Genetics..

[61] Jack CR, Knopman DS, Jagust WJ, Petersen RC, Weiner MW, Aisen PS (2013). Tracking pathophysiological processes in Alzheimer’s disease: an updated hypothetical model of dynamic biomarkers. Lancet Neurol..

[62] Maia LF, Kaeser SA, Reichwald J, Hruscha M, Martus P, Staufenbiel M, et al. Changes in amyloid-β and tau in the cerebrospinal fluid of transgenic mice overexpressing amyloid precursor protein. Sci Transl Med. 2013;5:194re192. 10.1126/scitranslmed.3006446 23863834

[63] Bright J, Hussain S, Dang V, Wright S, Cooper B, Byun T (2015). Human secreted tau increases amyloid-β production. Neurobiol Aging..

[64] Meredith JE, Sankaranarayanan S, Guss V, Lanzetti AJ, Berisha F, Neely RJ (2013). Characterization of novel CSF tau and ptau biomarkers for Alzheimer’s disease. PLoS One..

[65] Pooler AM, Phillips EC, Lau DH, Noble W, Hanger DP (2013). Physiological release of endogenous tau is stimulated by neuronal activity. EMBO Rep..

[66] Braak H, Braak E (1997). Frequency of stages of Alzheimer-related lesions in different age categories. Neurobiol Aging..

[67] Braak H, Zetterberg H, Del Tredici K, Blennow K (2013). Intraneuronal tau aggregation precedes diffuse plaque deposition, but amyloid-β changes occur before increases of tau in cerebrospinal fluid. Acta Neuropathol..

[68] Shen Y, Yang L, Li R (2013). What does complement do in Alzheimer’s disease? Old molecules with new insights. Transl Neurodegener..

[69] Yasojima K, Schwab C, McGeer EG, McGeer PL (1999). Up-regulated production and activation of the complement system in Alzheimer’s disease brain. Am J Pathol..

[70] Bonifati DM, Kishore U (2007). Role of complement in neurodegeneration and neuroinflammation. Mol Immunol..

[71] Wyss-Coray T, Yan F, Lin AH, Lambris JD, Alexander JJ, Quigg RJ (2002). Prominent neurodegeneration and increased plaque formation in complement-inhibited Alzheimer’s mice. Proc Natl Acad Sci U S A..

[72] Maier M, Peng Y, Jiang L, Seabrook TJ, Carroll MC, Lemere CA (2008). Complement C3 deficiency leads to accelerated amyloid β plaque deposition and neurodegeneration and modulation of the microglia/macrophage phenotype in amyloid precursor protein transgenic mice. J Neurosci..

[73] Fu H, Liu B, Frost JL, Hong S, Jin M, Ostaszewski B (2012). Complement component C3 and complement receptor type 3 contribute to the phagocytosis and clearance of fibrillar Aβ by microglia. Glia..

[74] Choucair-Jaafar N, Laporte V, Levy R, Poindron P, Lombard Y, Gies JP (2011). Complement receptor 3 (CD11b/CD18) is implicated in the elimination of β-amyloid peptides. Fundam Clin Pharmacol..

[75] Fonseca MI, Chu SH, Berci AM, Benoit ME, Peters DG, Kimura Y (2011). Contribution of complement activation pathways to neuropathology differs among mouse models of Alzheimer’s disease. J Neuroinflammation..

[76] d’Abramo C, Acker CM, Jimenez HT, Davies P. Tau passive immunotherapy in mutant P301L mice: c. 10.1371/journal.pone.0062402 PMC3639259 23638068

[77] Tatebayashi Y, Iqbal K, Grundke-Iqbal I (1999). Dynamic regulation of expression and phosphorylation of tau by fibroblast growth factor-2 in neural progenitor cells from adult rat hippocampus. J Neurosci..

